# Intestinal Insulin Signaling Encodes Two Different Molecular Mechanisms for the Shortened Longevity Induced by Graphene Oxide in *Caenorhabditis elegans*

**DOI:** 10.1038/srep24024

**Published:** 2016-04-04

**Authors:** Yunli Zhao, Ruilong Yang, Qi Rui, Dayong Wang

**Affiliations:** 1 Key Laboratory of Environmental Medicine Engineering in Ministry of Education, Medical School, Southeast University, Nanjing 210009, China; 2 Department of Preventive Medicine, Bengbu Medical College, Bengbu 233020, China; 3 College of Life Sciences, Nanjing Agricultural University, Nanjing 210095, China

## Abstract

Graphene oxide (GO) has been shown to cause multiple toxicities in various organisms. However, the underlying molecular mechanisms for GO-induced shortened longevity are still unclear. We employed *Caenorhabditis elegans* to investigate the possible involvement of insulin signaling pathway in the control of GO toxicity and its underlying molecular mechanisms. Mutation of *daf-2, age-1, akt-1*, or *akt-2* gene induced a resistant property of nematodes to GO toxicity, while mutation of *daf-16* gene led to a susceptible property of nematodes to GO toxicity, suggesting that GO may dysregulate the functions of DAF-2/IGF-1 receptor, AGE-1, AKT-1 and AKT-2-mediated kinase cascade, and DAF-16/FOXO transcription factor. Genetic interaction analysis suggested the involvement of signaling cascade of DAF-2-AGE-1-AKT-1/2-DAF-16 in the control of GO toxicity on longevity. Moreover, intestinal RNA interference (RNAi) analysis demonstrated that GO reduced longevity by affecting the functions of signaling cascade of DAF-2-AGE-1-AKT-1/2-DAF-16 in the intestine. DAF-16 could also regulate GO toxicity on longevity by functioning upstream of SOD-3, which encodes an antioxidation system that prevents the accumulation of oxidative stress. Therefore, intestinal insulin signaling may encode two different molecular mechanisms responsible for the GO toxicity in inducing the shortened longevity. Our results highlight the key role of insulin signaling pathway in the control of GO toxicity in organisms.

Carbon nanomaterials are a family of important engineered nanomaterials (ENMs) with many unique properties and potential applications^1^,^2^. Graphene oxide (GO) is a member of two-dimensional carbon ENMs with a single layer of sp^2^-bonded carbon atoms^3^. Due to its chemical stability, high coefficient of thermal conduction, amphipathicity, large surface area, and ease of functionalization, GO carries great potential for use in drug delivery, bioimaging, tissue engineering, and bioassay agents^1^,^2^.

Given the exceptional promise of GO applications, a number of studies with a focus on GO toxicity have been performed^4^. Some studies have suggested cytotoxicity and several aspects of adverse effects on animals such as pulmonary toxicity and immunotoxicity from GO exposure^4^,^5^,^6^,^7^,^8^. More recently, some reports have been published with the goal to further elucidate the molecular mechanisms of GO toxicity^9^,^10^,^11^,^12^. One study has demonstrated that activation of toll-like receptor 4 (TLR4) signaling and the subsequent autocrine TNF-α production might be involved in the control of GO toxicity in macrophages^10^. In addition, dysregulated mRNAs, proteins, and microRNAs (miRNAs) have been identified in GO exposed human HepG2 cells or GLC-82 cells^9^,^11^,^12^.

*Caenorhabditis elegans* is a classic animal model that offer an assay system for investigating the *in vivo* toxicological mechanisms of toxicants including the ENMs^13^. *C. elegans* share conserved properties for basic physiological processes, stress response, and molecular signaling pathways with humans^14^. Using *C. elegans*, previous studies have suggested the GO toxicity on the functions of both primary (such as intestine) and secondary (such as neuron and reproductive organs) targeted organs^15^,^16^,^17^,^18^. Regarding the cellular mechanisms of GO toxicity, the published data have suggested that GO toxicity might be consequent to the bioavailability, induction of reactive oxygen species (ROS), enhanced intestinal permeability, disrupted innate immune response, and prolonged defecation cycle length in nematodes^16^,^17^,^19^,^20^. Previous studies have also implied the crucial role of intestinal biological barrier against GO toxicity in nematodes^17^,^21^. It has also been reported that the induction of oxidative stress might play a key role in the chemical mechanism for GO induced shortened longevity in exposed nematodes^15^. However, the molecular mechanisms for the shortened longevity induced by GO toxicity are still largely unknown.

Insulin/insulin-like growth factor (IGF) signaling pathway has been implicated as a key molecular mechanism for longevity control in nematodes^22^,^23^. This signaling pathway has also been shown to play key roles in regulating other important biological processes including fat storage, innate immunity, and stress response^24^,^25^,^26^. In addition we have previously shown that insulin signaling regulates the toxicity of traffic-related particle matter (PM_2.5_) particles in *C. elegans*^26^. In *C. elegans*, insulin ligands bind to DAF-2/IGF-1 receptor (InR), activate tyrosine kinase activity, and initiate the cascade of several kinases: AGE-1/phosphatidiylinositol 3-kinase (PI3K), PDK-1/3-phosphoinositide-dependent kinase 1, AKT-1/2/serine/threonine kinase Akt/PKB, and SGK-1/serine or threonine-protein kinase^23^. AKT and SGK-1 further phosphorylate and inactivate the transcription factor DAF-16/FOXO, thereby blocking the transcription of target genes such as the *sod-3* gene^23^,^27^,^28^,^29^,^30^. DAF-18/phosphatidylinositol 3, 4, 5-triphosphate 3-phosphatase (PTEN) dephosphorylates AGE-1, which prevents DAF-16 phosphorylation by downstream kinases^23^,^27^,^29^. Tissue-specific activity assay has demonstrated the important functions of DAF-16 in regulating longevity in the intestine and neurons of nematodes^30^. Interestingly, our previous study has suggested that mutation of *sod-3* gene resulted in a susceptible property of nematodes to GO toxicity on lifespan, reproduction, and locomotion behavior, and induced a more severe induction of ROS production in GO exposed nematodes^19^,^21^. In the present study, we employed an *in vivo* assay system of *C. elegans* to investigate the possible involvement of insulin signaling pathway in the control of GO toxicity and its potential molecular mechanisms. Our results suggest that the insulin signaling pathway may encode two different molecular mechanisms for the shortened lifespan induced by GO in nematodes. The examination of these molecular mechanisms of the regulation of GO toxicity by the insulin signaling pathway provides an important basis for further elucidation of molecular networks involved in the control of GO toxicity in organisms.

## Results

### Physicochemical properties of GO

The size distribution of GO in the K-medium is shown in Fig. S1a. Most of the GO in K-medium were in the range of 40–50 nm (Fig. S1a). GO aggregation size was 386 ± 75 nm (Fig. S1a). GO appeared sheet-like, with a thickness of about 1.0 nm in topographic height, which corresponds to approximately one layer (Fig. S1b). Zeta potential of the GO (100 mg/L) in K-medium was −22.5 ± 1.7 mV. Raman spectroscopy measurement demonstrated a D-band signal of GO after treatment with sulfuric acid and KMnO_4_, indicating an introduction of disorder into the graphite layer (Fig. S1c). A G band and a D band were found at 1589 cm^−1^ and at 1336 cm^−1^, respectively (Fig. S1c).

### GO exposure influenced the expression patterns of genes that encode the insulin signaling pathway in nematodes

**Figure 1:**
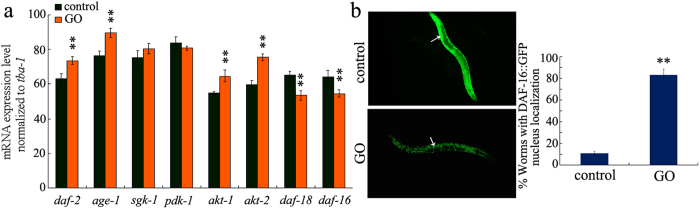
Effects of GO exposure on the expression patterns of genes encoding insulin signaling pathway in wild-type nematodes. (**a**) GO exposure altered expression levels of some genes encoding insulin signaling pathway in wild-type nematodes. (**b**) GO exposure influenced the nucleus translocation of DAF-16::GFP. Arrowheads indicate the DAF-16 expression in intestine. GO exposure concentration was 100 mg/L. Prolonged exposure was performed from L1-larvae to young adults. Bars represent means ± SEM. ^**^P < 0.01 *vs.* control.

Among the genes that encode the insulin signaling pathway in *C. elegans*, GO exposure (100 mg/L) resulted in a significant increase in the expression levels of *daf-2, age-1, akt-1*, and *akt-2* genes, and a decrease in the expression levels of *daf-18* and *daf-16* genes in wild-type nematodes (Fig. 1a). Moreover, considering the fact that the insulin signaling pathway can regulate some biological processes, such as longevity, through limiting DAF-16 nuclear localization^29^, we postulated that GO exposure might also affect the DAF-16 nuclear localization in nematodes. With the aid of transgenic strain of *zIs356,* we observed a significant increase in DAF-16:GFP expression in the nuclei of GO exposed (100 mg/L) nematodes compared with control (Fig. 1b). Thus, GO exposure may not only affect the transcriptional activities of genes encoding the insulin signaling pathway, but also influence the nucleus-cytoplasm translocation of DAF-16 in nematodes.

### Mutation of *daf-16* gene induced a susceptible property of nematodes to GO toxicity

**Figure 2:**
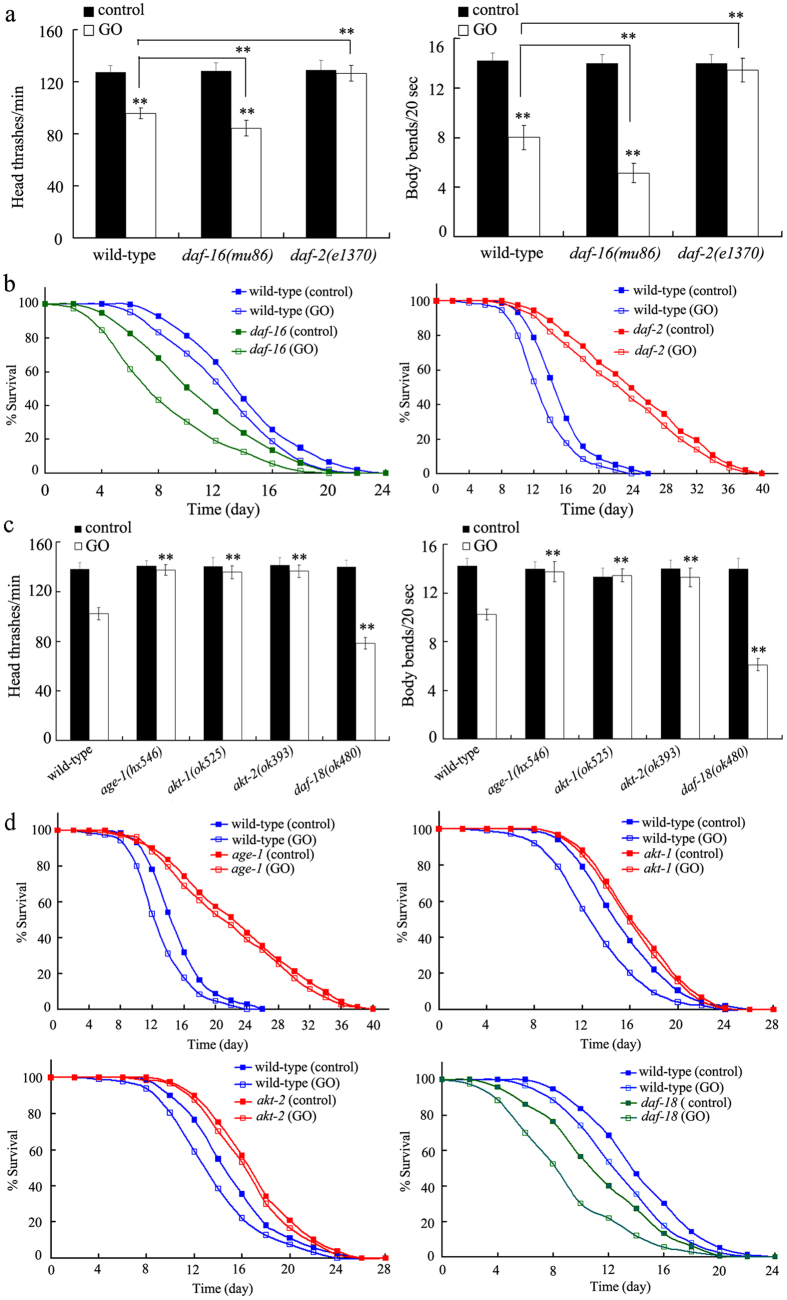
Effects of *daf-16, daf-2, age-1*, akt-1, *akt-2*, or *daf-18* mutation on nematodes exposed to GO. (**a**) Effects of *daf-16* or *daf-2* gene mutation on locomotion behavior in nematodes exposed to GO. (**b**) Effects of *daf-16* or *daf-2* gene mutation on lifespan in nematodes exposed to GO. (**c**) Mutations of *age-1, akt-1, akt-2*, or *daf-18* gene affected GO toxicity on locomotion behavior in nematodes. (**d**) Mutations of *age-1, akt-1, akt-2*, or *daf-18* gene affected GO toxicity on lifespan in nematodes. Locomotion behavior of nematodes was assessed by endpoints of head thrash and body bend. GO exposure concentration was 100 mg/L. Prolonged exposure was performed from L1-larvae to young adults. Bars represent means ± SEM. ^**^P < 0.01 *vs.* control (if not specially indicated).

In *C. elegans*, the *daf-16* gene encodes a FOXO transcriptional factor, which regulates several biological processes by affecting the functions of its targeted genes^23^. Previous studies have implied that locomotion behavior is a very sensitive endpoint for assessing the adverse effects of ENMs in nematodes^31^,^32^. Locomotion behavior was evaluated by the head thrash and body bend in nematodes^31^,^32^. After prolonged exposure, we found that loss-of-function mutation of *daf-16* gene resulted in more severe decrease in head thrash or body bend in GO-exposed nematodes compared with GO-exposed wild-type nematodes (Fig. 2a). Using lifespan as the endpoint, we further observed that GO-exposed *daf-16*(*mu86*) mutants showed more severely reduced lifespan compared with GO-exposed wild-type nematodes (Fig. 2b, Table S1). These results suggest that mutation of *daf-16* gene induces higher susceptibility to GO toxicity in *C. elegans*.

### Mutation of *daf-2* gene induced a resistant property of nematodes to GO toxicity

In *C. elegans*, the *daf-2* gene encodes an insulin receptor, which is responsible for receiving signals from the insulin peptides^23^. Using locomotion behavior as the endpoint, we found that *daf-2*(*e1370*) mutants were resistant to GO toxicity on both the head thrash and the body bend compared with GO-exposed wild-type nematodes (Fig. 2a). Moreover, GO-exposed *daf-2*(*e1370*) mutants exhibited significantly increased lifespan compared with GO-exposed wild-type nematodes (Fig. 2b, Table S1). Therefore, our results suggest that the *daf-2*(*e1370*) mutation induces resistance to GO toxicity.

### *age-1, akt-1, akt-2*, or *daf-18* genes were involved in the control of GO toxicity in nematodes

In *C. elegans*, the *age-1, akt-1*, and *akt-2* genes encodes the kinase cascade between the DAF-2 and the DAF-16, and DAF-18 acts as a suppressor of the AGE-1 in the insulin signaling pathway^23^. Using locomotion behavior and lifespan as the endpoints, we found that *age-1*(*hx546*), *akt-1*(*ok525*), and *akt-2*(*ok393*) mutants were resistant to GO toxicity on locomotion behavior or lifespan (Fig. 2c,d, Table S2). In contrast, *daf-18*(*ok480*) mutants were susceptible to GO toxicity on locomotion behavior or lifespan (Fig. 2c,d, Table S2). These results suggest that AGE-1, AKT-1, and AKT-2 constitute a kinase cascade for insulin signaling pathway, which is involved in the control of GO toxicity in nematodes.

### Genetic interactions of gene in the insulin signaling pathway in regulating GO toxicity on longevity in nematodes

**Figure 3:**
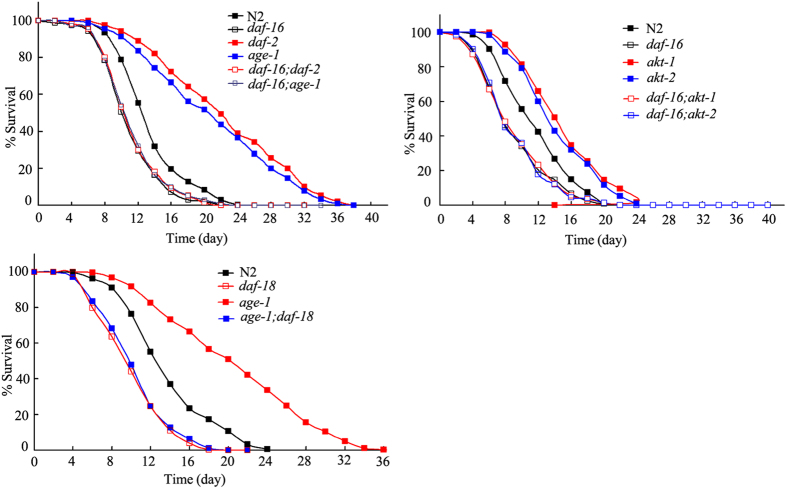
Genetic interactions of genes in the insulin signaling pathway in regulating the GO toxicity on lifespan in nematodes. GO exposure concentration was 100 mg/L. Prolonged exposure was performed from L1-larvae to young adults.

We next investigated the genetic interactions of the *daf-16* gene with other genes in the insulin signaling pathway in regulating GO toxicity on longevity in nematodes. We found that mutation of the *daf-16* gene could effectively reduce the lifespan in GO-exposed *daf-2*(*e1370*), *age-1*(*hx546*), *akt-1*(*ok525*), or *akt-2*(*ok393*) mutants (Fig. 3, Table S3). These results imply that DAF-16 may act downstream of DAF-2, AGE-1, AKT-1, and AKT-2 to regulate GO toxicity on longevity in nematodes.

We further found that mutation of the *daf-18* gene could effectively reduce the lifespan in GO-exposed *age*(*hx546*) mutants (Fig. 3, Table S3), which confirms the suppressor role of DAF-18 on AGE-1 in the control of GO toxicity in nematodes.

### Tissue-specific activity of DAF-16 in regulating the GO toxicity in nematodes

**Figure 4:**
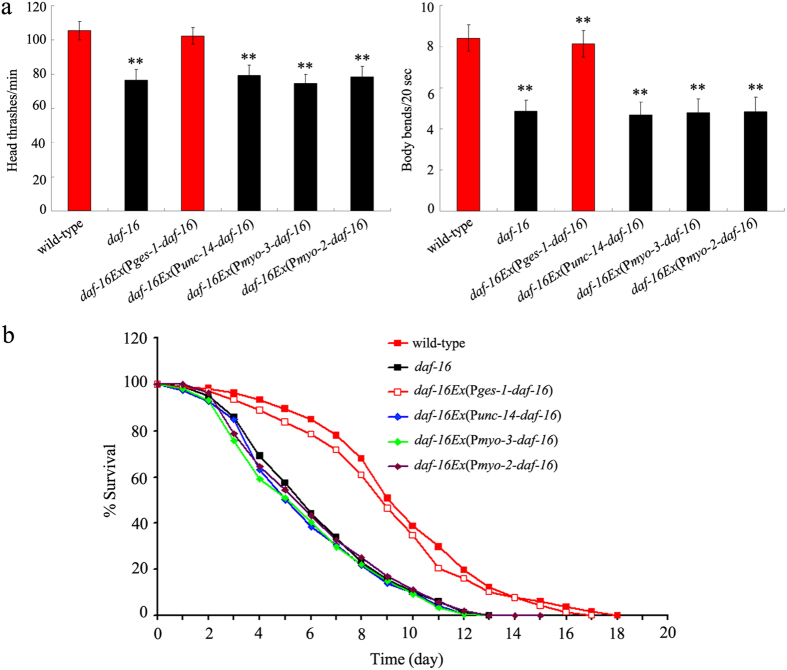
Tissue-specific activity of DAF-16 in regulating the GO toxicity in nematodes. (**a**) Tissue-specific activity of DAF-16 in regulating GO toxicity on locomotion behavior in nematodes. Locomotion behavior of nematodes was assessed by endpoints of head thrash and body bend. (**b**) Tissue-specific activity of DAF-16 in regulating GO toxicity on lifespan in nematodes. GO exposure concentration was 100 mg/L. Prolonged exposure was performed from L1-larvae to young adults. Bars represent means ± SEM. ^**^P < 0.01 *vs.* wild-type.

In *C. elegans*, the *daf-16* gene is expressed in almost all tissues including the intestine, neurons, muscle, and pharynx^23^. Using locomotion behavior and lifespan as the endpoints, we found that expression of *daf-16* gene in the neurons, muscle, or pharynx did not significantly influence the locomotion behavior or lifespan in GO-exposed *daf-16*(*mu86*) mutants (Fig. 4, Table S4). In contrast, we observed that expression of *daf-16* gene in the intestine effectively augmented the decreased locomotion behavior or reduced lifespan in GO-exposed *daf-16*(*mu96*) mutants (Fig. 4, Table S4). These results suggest that the *daf-16* gene may act primarily in the intestine to regulate the GO toxicity in nematodes.

### Intestine-specific RNA interference (RNAi) of genes encoding the insulin signaling pathway affected GO toxicity on longevity in nematodes

**Figure 5:**
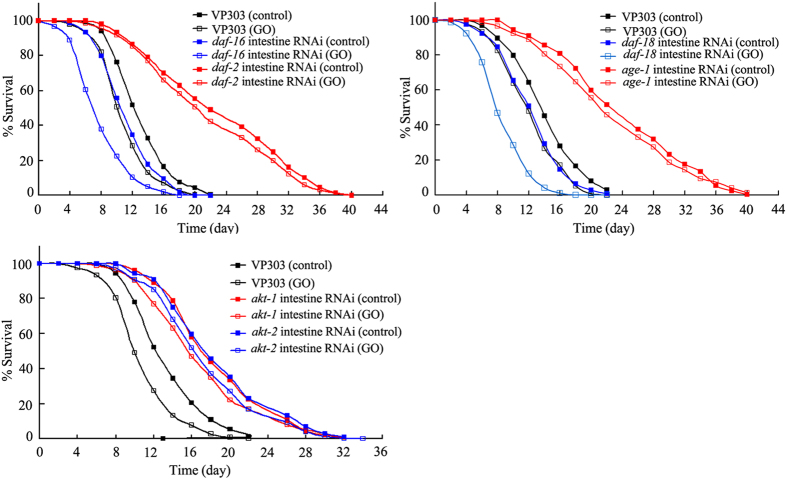
Effects of intestine-specific RNAi of the genes encoding insulin signaling pathway on lifespan in GO exposed nematodes. GO exposure concentration was 100 mg/L. Prolonged exposure was performed from L1-larvae to young adults.

To further examine the tissue-specific activity of other genes in the insulin signaling pathway in regulating GO toxicity, we performed intestine-specific RNAi on the VP303 strain^33^. In nematodes, we found that intestine-specific RNAi of the *daf-2, age-1, akt-1*, or *akt-2* gene resulted in prolonged lifespan, whereas intestine-specific RNAi of the *daf-16* or *daf-18* gene led to reduced lifespan (Fig. 5, Table S5). Moreover, after prolonged exposure, a resistant property to GO toxicity was observed in nematodes with the intestine-specific RNAi of the *daf-2, age-1, akt-1*, or *akt-2* gene, whereas a susceptible property to GO toxicity was observed in nematodes subjected to intestine-specific RNAi of the *daf-16* or *daf-18* gene (Fig. 5, Table S5). These results suggest that the insulin signaling pathway can act in the intestine to regulate GO toxicity in nematodes. Since the VP303 strain exhibits deficits in locomotion behavior, we did not examine the effects of intestine-specific RNAi of genes encoding the insulin signaling pathway on locomotion behavior in GO-exposed nematodes.

### Regulation of GO toxicity by the DAF-16 target SOD-3

**Figure 6:**
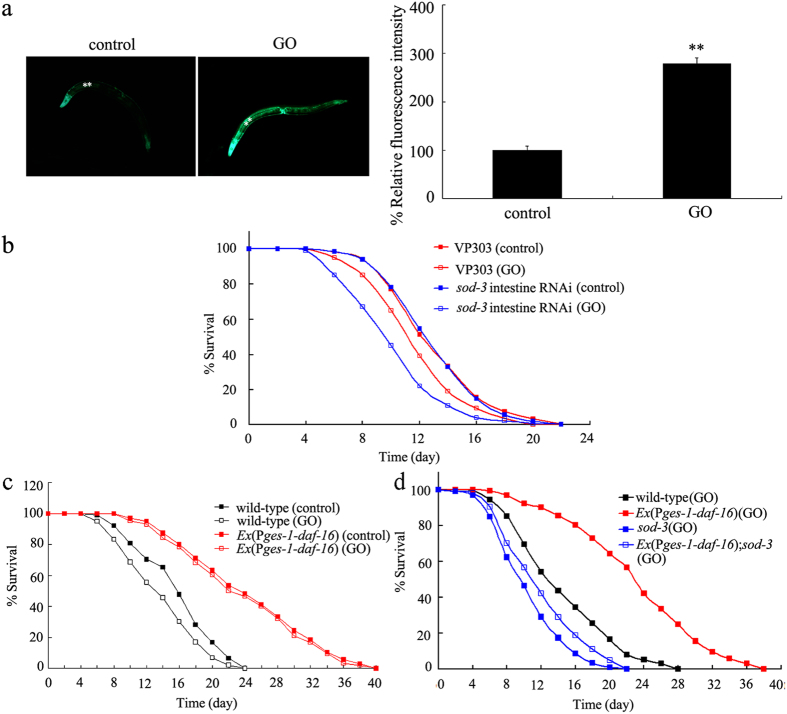
Role of SOD-3 in regulating the GO toxicity in nematodes. (**a**) Effects of GO exposure on SOD-3::GFP expression. The left shows images for SOD-3::GFP expression, and the right shows comparison of relative fluorescence intestine of SOD-3::GFP in intestine of nematodes. Asterisks indicate the intestine of nematodes. (**b**) Effects of intestine-specific RNAi of *sod-3* gene on lifespan in GO exposed nematodes. (**c**) Effects of intestinal overexpression of *daf-16* gene on GO toxicity on lifespan in nematodes. (**d**) Effects of *sod-3* mutation on lifespan in GO-exposed nematodes overexpressing *daf-16* gene in intestine in nematodes. GO exposure concentration was 100 mg/L. Prolonged exposure was performed from L1-larvae to young adults. Bars represent means ± SEM. ^**^P < 0.01 *vs.* wild-type.

In *C. elegans, sod-3* is an important target gene of the *daf-16* gene, and it encodes a mitochondrial iron/manganese superoxide dismutase required for defending against oxidative stress^23^. SOD-3 is expressed in the pharynx in the head, intestine, muscle, vulva, and tail^34^. In general, weak expression of SOD-3 is expected in the intestine^34^. After prolonged exposure, however, we observed that GO could significantly increase the expression of SOD-3 in the intestine of nematodes compared with that of control (Fig. 6a).

Using VP303 as the intestinal RNAi tool, we found that intestinal RNAi of the *sod-3* gene induced a susceptible property of nematodes to GO toxicity on longevity in nematodes (Fig. 6b, Table S6). Intestinal RNAi of the *sod-3* gene, however, did not affect the lifespan in nematodes in the absence of GO exposure (Fig. 6b, Table S6). Therefore, SOD-3 may also be able to act in the intestine to regulate GO toxicity on longevity in nematodes.

### Genetic interaction between DAF-16 and SOD-3 in regulating GO toxicity on longevity in nematodes

To further examine the interaction between the *daf-16* gene and the *sod-3* gene in regulating GO toxicity, we constructed the transgenic strain of *Ex*(*Pges-1-daf-16*), in which the *daf-16* gene was overexpressed in the intestine of nematodes. Intestinal overexpression of the *daf-16* gene induced a resistant property of animals to GO toxicity on longevity (Fig. 6c, Table S7). In contrast, we observed that the resistant property of the transgenic strain of *Ex*(*Pges-1-daf-16*) to GO toxicity on longevity could be noticeably inhibited by *sod-3* mutation in nematodes (Fig. 6d, Table S7). Under normal conditions, the *sod-2*(*gk235*) mutant had similar lifespan to that in wild-type N2, whereas mutation of the *sod-2* gene did not obviously affect the long-lived phenotype of nematodes overexpressing the *daf-16* gene in intestine (Fig. S2, Table S7). These results suggest that DAF-16 may further function upstream of SOD-3 to regulate GO toxicity on longevity in nematodes.

### Genetic interaction between DAF-16 and SOD-3 in regulating GO toxicity in inducing ROS production

Finally, we investigated the interaction between DAF-16 and SOD-3 in regulating GO toxicity in inducing intestinal ROS production in nematodes. Mutation of the *sod-3* gene or overexpression of the *daf-16* gene in intestine did not induce significant ROS production in nematodes in the absence of GO exposure (Fig. S3). However, after GO exposure, we observed that overexpresion of the *daf-16* gene in the intestine significantly suppressed the induction of intestinal ROS production in nematodes (Fig. S3). In contrast, after GO exposure, mutation of the *sod-3* gene strengthened the induction of intestinal ROS production in nematodes (Fig. S3). Moreover, after GO exposure, mutation of the *sod-3* gene further induced significant induction of intestinal ROS production in nematodes overexpressing the *daf-16* gene in intestine (Fig. S3).

## Discussion

**Figure 7:**
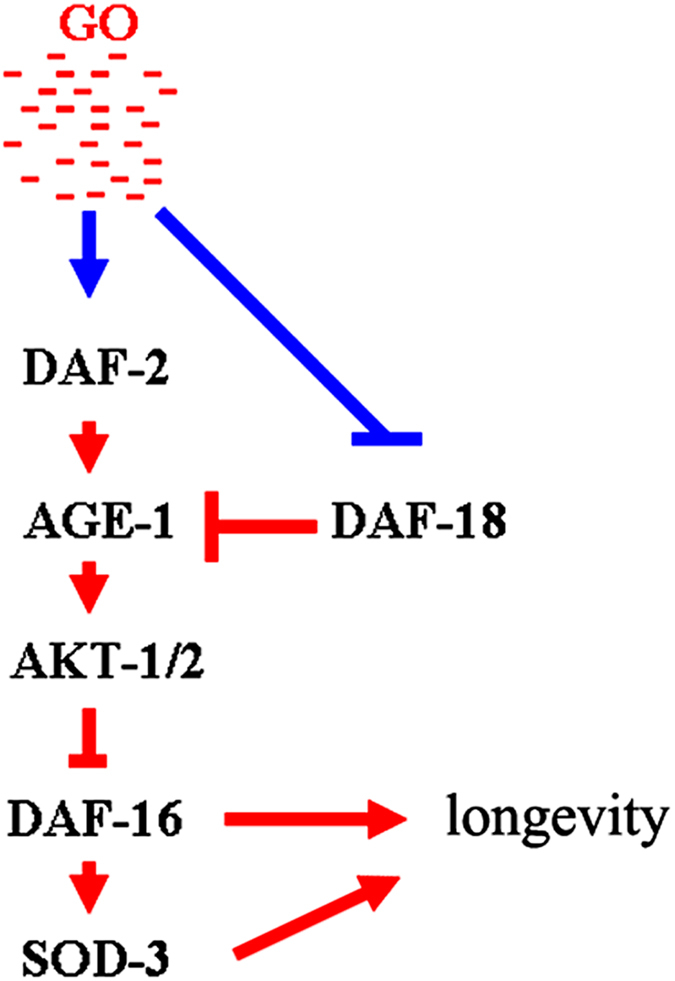
A model for the insulin signaling pathway in the control of GO toxicity in nematodes.

Previous studies have suggested that the insulin signaling pathway is involved in the control of biological processes such as the innate immunity and stress response in nematodes^25^,^26^. In the present study, we further provided evidence to support the involvement of the insulin signaling pathway in the control of toxicity of specific ENMs such as GO. In nematodes, GO exposure at the concentration of 100 mg/L increased the expression levels of the *daf-2, age-1, akt-1*, and *akt-2* genes, whereas decreased the expression levels of the *daf-18* and *daf-16* genes (Fig. 1a). These results imply that, at least at the examined concentration, GO exposure may dysregulate functions of the DAF-2/IGF-1 receptor, AGE-1, AKT-1 and AKT-2-mediated kinase cascade, and DAF-16/FOXO transcription factor (Fig. 7). More interestingly, we also found that GO is capable of affecting the function of AGE-1 through downregulating the expression of DAF-18 in nematodes (Fig. 7).

Evidence from the genetic mutants further confirmed the roles of DAF-2, AGE-1, AKT-1, AKT-2, DAF-18, and DAF-16 in regulating GO toxicity. Mutation of the *daf-2, age-1, akt-1*, or *akt-2* gene induced a resistant property of nematodes to GO toxicity; however, mutation of *daf-18* or *daf-16* gene induced a susceptible property of nematodes to GO toxicity (Fig. 2, Table S1 and S2). In *C. elegans,* it has been reported that the *daf-2, age-1, akt-1*, and *akt-2* mutants were resistant to infection of *Pseudomonas aeruginosa*; however, the *P. aeruginosa*-infected *daf-18* or *daf-16* mutant showed similar lifespan to that of *P. aeruginosa*-infected wild-type nematodes^25^. It has been further reported that PM_2.5_ significantly increased the expression levels of the *daf-2* and *akt-1* genes, while decreased the expression levels of the *daf-18* and *daf-16* genes^26^. These results imply that the insulin signaling pathway may regulate different biological processes through different signaling cascade in nematodes.

Genetic interaction analysis has suggested that basic signaling cascade of DAF-2-AGE-1-AKT-1/2-DAF-16 might play a role in the control of GO toxicity on longevity in nematodes (Figs 3 and 7, Table S3). The signaling cascade of DAF-2-AGE-1-AKT-1/2-DAF-16 has also been shown to be involved in the control of immune response of nematodes in the context of *P. aeruginosa* infection^25^. These results suggest that DAF-2-AGE-1-AKT-1/2-DAF-16 may be a conserved signaling cascade for insulin signaling pathway in regulating biological events associated with innate immunity, stress response, and nanotoxicity in nematodes.

Previous studies have suggested that intestinal expression of DAF-16 largely accounts for its function in regulating longevity in nematodes^22^,^23^,^30^. However, the tissue-specific activity of DAF-16 in regulating stress response or nanotoxicity remains unclear. Furthermore, previous study has demonstrated that insulin signaling is involved in the control of PM_2.5_ toxicity on the development and function of intestine in nematodes^26^. In this study, we found that intestine-specific activity of DAF-16 was required for its function in regulating GO toxicity on longevity in nematodes (Fig. 4, Table S4). Interestingly, we observed that intestinal RNAi of genes encoding the signaling cascade of DAF-2-AGE-1-AKT-1/2-DAF-16 also affected GO toxicity on longevity in nematodes (Fig. 5, Table S5). These results imply that GO exposure may reduce longevity by dysregulating the functions of signaling cascade of DAF-2-AGE-1-AKT-1/2-DAF-16, a conserved signaling cascade required for the longevity control, in intestine of nematodes^22^,^23^. In other word, intestinal insulin signaling is likely involved in regulateing GO toxicity on longevity in nematodes. We hypothesize that altered insulin signaling might be one of the molecular mechanisms of GO toxicity on longevity in nematodes (Fig. 7).

In this study, we found that insulin signaling could potentially contribute to another molecular mechanism for GO toxicity on longevity. Our results suggest that the DAF-16 functioned upstream of SOD-3 to regulate GO toxicity on longevity (Fig. 6d, Table S7). In nematodes, the *sod-3* gene encodes the antioxidation system against the induction of oxidative stress. In GO-exposed nematodes, function of SOD-3 against oxidative stress was further confirmed by ROS production assay in *sod-3* mutant (Fig. S3). Interestingly, we found that mutation of the *sod-3* gene could further induce significant induction of ROS production in GO-exposed nematodes overexpressing the *daf-16* gene in intestine (Fig. S3). These results imply that GO exposure may also reduce longevity by affecting the functions of the antioxidation system against the induction of oxidative stress. We hypothesize that this is another molecular mechanism for GO toxicity on longevity, which is dependent of the insulin signaling and its important target gene *sod-3* in nematodes (Fig. 7). This proposed mechanism also provides basis for the role of oxidative stress in inducing GO toxicity in nematodes as described previously in other reports^15^.

In *C. elegans*, the DAF-16 acts as a transcription factor to activate the activity of its targeted genes when DAF-16 is translocated into the nucleus^22^,^23^. Interestingly, we observed that, along with the severely decreased expression of DAF-16, a certain amount of DAF-16 was found to be translocated into the nucleus (Fig. 1b). These results imply that at least a certain amount of DAF-16 translocated into the nucleus may also induce a positive feedback loop between DAF-16 and other components of the signaling pathway in nematodes.

In conclusion, we investigated the involvement of insulin signaling pathway in the control of GO toxicity on longevity and the underlying mechanisms in nematodes. We identified a signaling cascade of DAF-2-AGE-1-AKT-1/2-DAF-16 in the insulin signaling pathway that is involved in the control of GO toxicity on longevity. Moreover, we found that this signaling cascade acted primarily in the intestine to regulate the GO toxicity on longevity. Based on our results, we hypothesize that one of the molecular mechanism for GO toxicity is that GO could dysregulate the signaling cascade of DAF-2-AGE-1-AKT-1/2-DAF-16 in the insulin signaling pathway. Furthermore, we found that insulin signaling pathway could encode another molecular mechanism for the reduced longevity induced by GO. GO exposure could suppress the function of DAF-16 within the insulin signaling, and thereby, lead to further inhibition of the function of SOD-3, which plays an important role in defense against oxidative stress in GO exposed nematodes. The elucidated insulin signaling-encoded molecular mechanisms for the reduced longevity induced by GO highlight the potential role of insulin signaling pathway in the control of GO toxicity in organisms.

## Methods

### Reagents and preparation of GO

GO was prepared from the natural graphite powder using a modified Hummer’s method^35^,^36^. Graphite (2 g) and sodium nitrate (1 g) were added into a 250-mL flask. After addition of concentrated H_2_SO_4_ (50 mL) on ice, KMnO4 (7 g) was added to the mixture. And then, 90 mL of H_2_O was slowly dripped into the paste after the temperature of mixture warmed to 35 °C. After stirring diluted suspension at 70 °C for another 15 min, suspension was treated with the mixture of 7 mL of 30% H_2_O_2_ and 55 mL of H_2_O. The resulting warm suspension was filtered to obtain a yellow-brown filter cake, which was further washed with a solution of 3% HCl, followed by drying at 40 °C for 24 h. GO was obtained by ultrasonication of as-made graphite oxide in water for 1 h.

GO was sonicated for 30-min (40 kHz, 100 W), and dispersed in K medium to prepare a stock solution (1 mg/L). Stock solution was diluted to the used concentration (100 mg/L) with K medium just prior to exposure. Previous study has suggested that GO at the concentration of 100 mg/L could decrease lifespan in nematodes^16^. All the other chemicals were obtained from Sigma-Aldrich (St. Louis, MO, USA).

### Characterization of GO

GO was characterized by transmission electron microscopy (TEM, JEM-200CX, JEOL, Japan), atomic force microscopy (AFM, SPM-9600, Shimadzu, Japan), Raman spectroscopy using 632 nm wavelength excitation (Renishaw Invia Plus laser Raman spectrometer, Renishaw, UK), and zeta potential analyzed by Nano Zetasizer using a dynamic light scattering technique. To perform AFM measurement, GO suspension was pipetted on Si substrates, and the Si substrates were further air-dried and placed under AFM tip.

### *C. elegans* strain preparation

Nematodes were maintained on nematode growth medium (NGM) plates seeded with *Escherichia coli* OP50 at 20 °C as described previously^37^. Nematodes used in the present study were wild-type N2, mutants of *daf-16*(*mu86*), *daf-2*(*e1370*), *age-1*(*hx546*), *akt-1*(*ok525*), *akt-2*(*ok393*), *daf-18*(*ok480*), *sod-3*(*gk235*), *daf-16*(*mu86*);*sod-3*(*gk235*), and *daf-16-*(*mu86*);*daf-2*(*e1370*), and transgenic strains of VP303/*kbIs7*[*nhx-2p::rde-1*], *zIs356*[P*daf-16::daf-16a/b::GFP*], *daf-16*(*mu86*)*Ex*(P*ges-1-daf-16*), *daf-16*(*mu86*)*Ex*(P*unc-14-daf-16*), *daf-16*(*mu86*)*Ex*(P*myo-3-daf-16*), *daf-16*(*mu86*)*Ex*(P*myo-2-daf-16*), *Ex*(P*ges-1-daf-16*), and *Ex*(P*ges-1-daf-16*);*sod-3*(*gk235*). Some of the used strains were originally obtained from the *Caenorhabditis* Genetics Center (funded by NIH Office of Research Infrastructure Programs (P40 OD010440)). Gravid nematodes were washed off the plates into centrifuge tubes, and lysed with a bleaching mixture (0.45 M NaOH, 2% HOCl). Age synchronous populations of L1-larvae were prepared as described previously^38^. Prolonged exposure to GO was performed from L1-larvae to young adults in 12-well sterile tissue culture plates at 20 °C in the presence of food (OP50). The exposed nematodes were used for toxicity assessment using lifespan, locomotion behavior, and ROS production as the endpoints.

### Reverse-transcription and quantitative real-time polymerase chain reaction (qRT-PCR)

Total RNAs were extracted using RNeasy Mini kit (Qiagen), and then reverse transcribed using the PrimeScript^TM^ RT reagent kit (Takara, Otsu, Shiga, Japan). After cDNA synthesis, real-time PCR was performed using SYBR Premix Ex Taq™ (Takara) for amplification of PCR products. Quantitative reverse transcription PCR was run at the optimized annealing temperature of 58 °C. Relative quantification of the targeted genes in comparison to the reference *tba-1* gene encoding a tubulin protein was determined. The final results were expressed as the relative expression ratio between the targeted gene and the reference gene. The designed primers for targeted genes and reference *tba-1* gene were shown in Table S8. All reactions were performed in triplicate.

### DAF-16 nuclear translocation assay

DAF-16 nuclear translocation was scored after exposure the transgenic strain of *zIs356* to GO. Intestinal DAF-16::GFP was scored as nuclear localization in anterior part of the body. Images were taken with a Zeiss Imager A2 fluorescence microscope. Three independent experiments with ten nematodes per treatment were examined.

### Toxicity assessment

Lifespan assay was performed at 20 °C basically as described^39^. Hermaphrodite nematodes were transferred daily for the first 4 days of adulthood. After that, the nematodes were checked every day. Nematodes would be scored as dead if they did not move even after repeated taps with a pick. Forty nematodes were examined per treatment, and three replicates were performed.

Locomotion behavior of nematodes was assessed by the endpoints of head thrash and body bend as described^40^. A head thrash was defined as a change in the direction of bending at the mid body. A body bend was counted as a change in the direction of the part of the nematodes corresponding to the posterior bulb of the pharynx along the *y* axis, assuming that nematode was traveling along the *x* axis. Twenty nematodes were examined per treatment, and ten replicates were performed.

The method for ROS production was performed as described previously^41^. After exposure, nematodes were transferred to 1 μM 5′, 6′-chloromethyl-2′, 7′-dichlorodihydro-fluorescein diacetate (CM-H2DCFDA; Molecular Probes) in 12-well sterile tissue culture plates to incubate for 3 h at 20 °C in the dark without addition of food. Nematodes were mounted on 2% agar pads for examination at 488 nm of excitation wavelength and 510 nm of emission filter with a laser scanning confocal microscope (Leica, TCS SP2, Bensheim, Germany). Relative fluorescence intensity of intestine was semi-quantified. Semiquantified ROS was expressed as relative fluorescence units (RFU) and normalized to the autofluorescence. Thirty nematodes were examined per treatment, and five replicates were performed.

### RNAi

RNAi was performed by feeding nematodes with *E. coli* strain HT115 (DE3) expressing double-stranded RNA that is homologous to a target gene as described^42^. *E. coli* HT115 (DE3) grown in LB broth containing ampicillin (100 μg/mL) at 37 °C overnight was plated onto NGM containing ampicillin (100 μg/mL) and isopropyl 1-thio-β-D-galactopyranoside (IPTG, 5 mM). L2 larvae were placed on RNAi plates for 2 days at 20 °C until nematodes became gravid. Gravid adults were transferred to fresh RNAi-expressing bacterial lawns to lay eggs for 2 h so as to obtain the second generation of RNAi population. Eggs were allowed to develop at 20 °C to young adults for the subsequent assays.

### DNA constructs and germline transformation

To generate entry vector carrying promoter sequence, promoter regions for *ges-1* gene specially expressed in the intestine, *unc-14* gene specially expressed in neurons, *myo-3* gene specially expressed in muscle, and *myo-2* gene specially expressed in pharynx were amplified by PCR from wild-type *C. elegans* genomic DNA. The promoter fragments were inserted into the pPD95_77 vector in the sense orientation. *daf-16* cDNA was amplified by PCR, and inserted into corresponding entry vector carrying the *ges-1, unc-14, myo-3*, or *myo-2* promoter sequence. Germline transformation was performed as described by coinjecting the testing DNA at a concentration of 10–40 μg/mL and the marker DNA of P*lin-44::gfp* or *unc-119*(+) at a concentration of 60 μg/mL into the gonad of nematodes^43^.

### Statistical analysis

All data in this article were expressed as means ± standard error of the mean (S.E.M.). Graphs were generated using Microsoft Excel (Microsoft Corp., Redmond, WA). Statistical analysis was performed using SPSS 12.0 (SPSS Inc., Chicago, USA). Differences between groups were determined using analysis of variance (ANOVA). Probability levels of 0.05 and 0.01 were considered statistically significant.

## Additional Information

**How to cite this article**: Zhao, Y. *et al*. Intestinal Insulin Signaling Encodes Two Different Molecular Mechanisms for the Shortened Longevity Induced by Graphene Oxide in *Caenorhabditis elegans. Sci. Rep.* **6**, 24024; doi: 10.1038/srep24024 (2016).

## Supplementary Material

Supplementary Information
