# Supplementary material for: Intestinal Insulin Signaling Encodes Two Different Molecular Mechanisms for the Shortened Longevity Induced by Graphene Oxide in Caenorhabditis elegans
Source: Sci Rep. 2016 Apr 4;6:24024. doi: 10.1038/srep24024 (PMC4819199; doi:10.1038/srep24024)
Supplement: Supplementary Information [file srep24024-s1.pdf]

Intestinal Insulin Signaling Encoded Two Different Molecular Mechanisms for the Shortened Longevity Induced by Graphene Oxide in *Caenorhabditis elegans*

Yunli Zhao,[a, b] Ruilong Yang,[a, c] Qi Rui[c] & Dayong Wang[*a]

[a]Key Laboratory of Environmental Medicine Engineering in Ministry of Education, Medical School, Southeast University, Nanjing 210009, China

[b]Department of Preventive Medicine, Bengbu Medical College, Bengbu 233020, China

[c]College of Life Sciences, Nanjing Agricultural University, Nanjing 210095, China

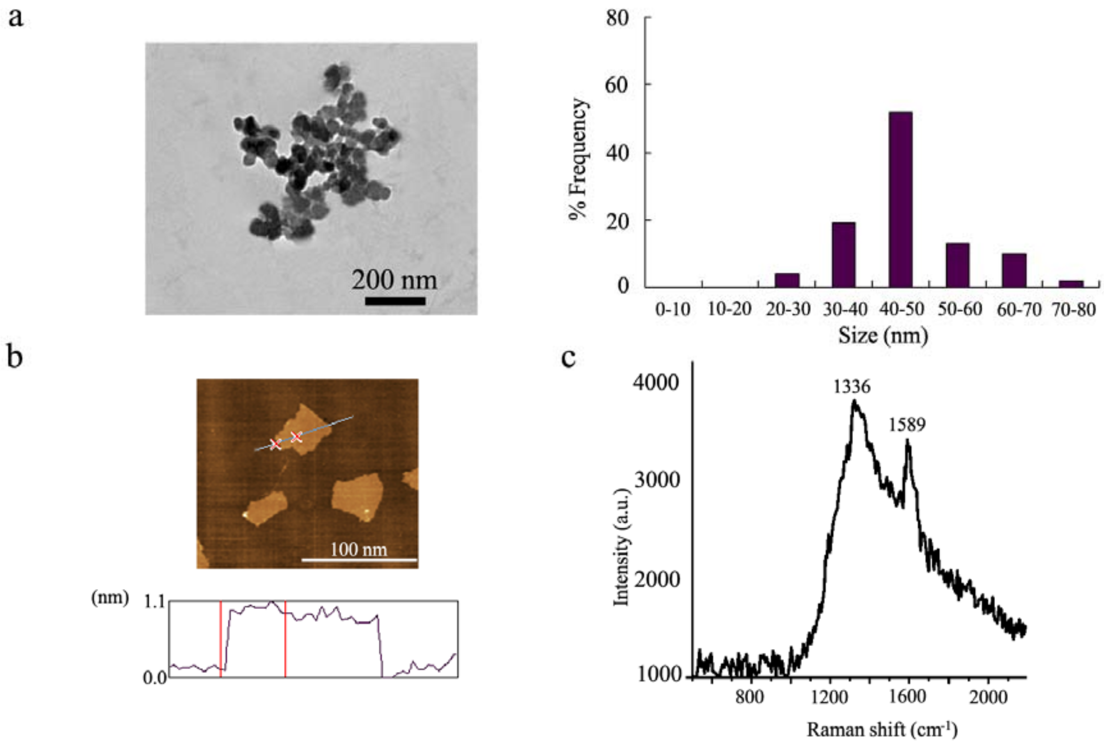

Figure S1 | Physiochemical properties of GO. (a) TEM of GO in K-medium. (b) AFM analysis of GO. (c) Raman spectrum of GO.

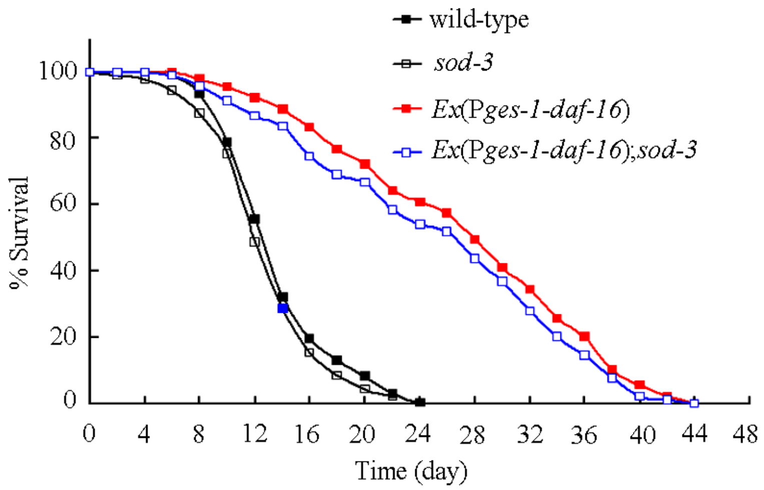

Figure S2 | Effects of *sod-3* mutation on lifespan in nematodes overexpressing *daf-16* gene in intestine in nematodes. Nematodes were not exposed to GO.

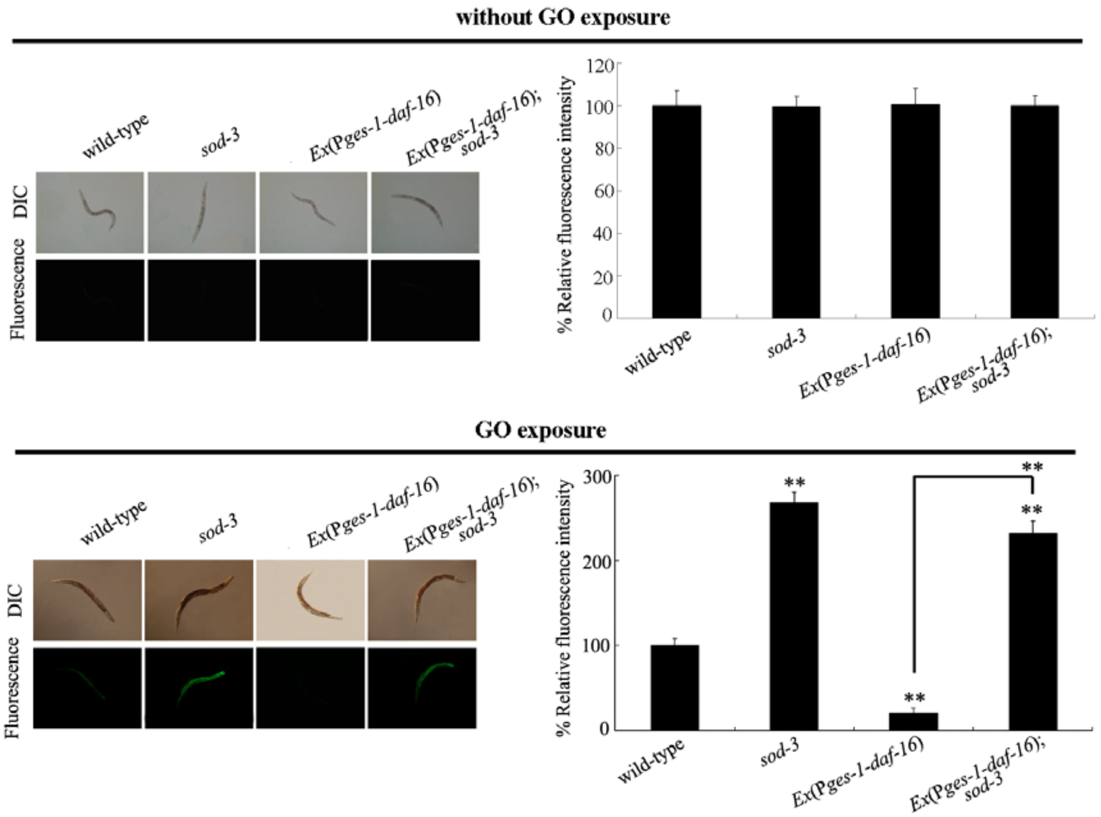

Figure S3 | Mutation of *sod-3* gene induced the induction of ROS production in GO-exposed nematodes overexpressing *daf-16* gene in intestine. GO exposure concentration was 100 mg L^{-1}. Prolonged exposure was performed from L1-larvae to young adults. Bars represent means ± SEM. $^{**}P < 0.01$ *vs.* wild-type (if not specially indicated).

Table S1 | Lifespan in *daf-16* or *daf-2* mutants exposed to GO

Treatment	Mean lifespan (day)	Significance (compared with N2)
Wild-type (control)	15.1 ± 0.6	
Wild-type (100 mg L^{-1} GO)	13.2 ± 0.4	
daf-16(mu86) (control)	10.8 ± 0.4	$P < 0.01$
daf-16(mu86) (100 mg L^{-1} GO)	6.5 ± 0.6	$P < 0.01$
Wild-type (control)	15.2 ± 0.6	
Wild-type (100 mg L^{-1} GO)	12.7 ± 0.8	
daf-2(e1370) (control)	23.9 ± 0.7	$P < 0.01$
daf-2(e1370) (100 mg L^{-1} GO)	22.6 ± 0.5	$P < 0.01$

Table S2 | Mutations of *age-1*, *akt-1*, *akt-2*, or *daf-18* gene affected the GO toxicity on lifespan in nematodes

Treatment	Mean lifespan (day)	Significance (compared with control)
Wild-type (control)	15.4 ± 0.6	
Wild-type (100 mg L^{-1} GO)	12.5 ± 0.8	
age-1(hx546) (control)	23.4 ± 0.5	$P < 0.01$
age-1(hx546) (100 mg L^{-1} GO)	21.3 ± 0.9	$P < 0.01$
Wild-type (control)	15.3 ± 0.7	
Wild-type (100 mg L^{-1} GO)	12.7 ± 0.3	
akt-1(ok525) (control)	17.1 ± 0.4	$P < 0.01$
akt-1(ok525) (100 mg L^{-1} GO)	16.6 ± 0.3	$P < 0.01$
Wild-type (control)	15.2 ± 0.5	
Wild-type (100 mg L^{-1} GO)	12.4 ± 0.6	
akt-2(ok393) (control)	17.4 ± 0.6	$P < 0.01$
akt-2(ok393) (100 mg L^{-1} GO)	16.9 ± 0.4	$P < 0.01$
Wild-type (control)	15.1 ± 0.3	
Wild-type (100 mg L^{-1} GO)	12.7 ± 0.7	
daf-18(ok480) (control)	11.2 ± 0.6	$P < 0.01$
daf-18(ok480) (100 mg L^{-1} GO)	8.5 ± 0.5	$P < 0.01$

Table S3 | Genetic interactions of genes in the insulin signaling pathway in regulating GO toxicity on lifespan in nematode

Treatment	Mean lifespan (day)	Significance (compared with wild-type)
Wild-type	12.3 ± 0.5	
daf-16(mu86)	8.9 ± 0.7	$P < 0.01$
daf-2(e1370)	22.4 ± 0.6	$P < 0.01$
age-1(hx546)	21.7 ± 0.9	$P < 0.01$
daf-16(mu86);daf-2(e1370)	9.3 ± 0.7	$P < 0.01$
daf-16(mu86);age-1(hx546)	9.8 ± 0.9	$P < 0.01$
Wild-type	12.4 ± 0.6	
daf-16(mu86)	8.2 ± 0.8	$P < 0.01$
akt-1(ok525)	15.9 ± 0.5	$P < 0.01$
akt-2(ok393)	14.9 ± 0.4	$P < 0.01$
daf-16(mu86);akt-1(ok525)	8.7 ± 0.6	$P < 0.01$
daf-16(mu86);akt-2(ok393)	8.3 ± 0.7	$P < 0.01$
Wild-type	12.5 ± 0.8	
daf-18(ok480)	9.1 ± 0.5	$P < 0.01$
age-1(hx546)	21.5 ± 0.8	$P < 0.01$
age-1(hx546);daf-18(ok480)	9.2 ± 0.7	$P < 0.01$

GO exposure concentration was 100 mg L^{-1}.

Table S4 | Tissue-specific activity of DAF-16 in regulating toxicity of GO on lifespan in nematodes

Treatment	Mean lifespan (day)	Significance (compared with wild-type)
Wild-type (100 mg L^{-1} GO)	11.8 ± 0.7	
daf-16(mu86) (100 mg L^{-1} GO)	6.7 ± 0.8	$P < 0.01$
daf-16(mu86)Ex(P*ges-1-daf-16*) (100 mg L^{-1} GO)	11.1 ± 0.9	NS
daf-16(mu86)Ex(P*unc-14-daf-16*) (100 mg L^{-1} GO)	6.9 ± 1.1	$P < 0.01$
daf-16(mu86)Ex(P*myo-3-daf-16*) (100 mg L^{-1} GO)	6.3 ± 0.5	$P < 0.01$
daf-16(mu86)Ex(P*myo-2-daf-16*) (100 mg L^{-1} GO)	6.2 ± 0.7	$P < 0.01$

NS, no significance.

Table S5 | Effects of intestinal RNAi of genes encoding insulin signaling pathway on GO toxicity on lifespan in nematodes

Treatment	Mean lifespan (day)	Significance (compared with VP303)
VP303 (control)	12.9 ± 0.7	
VP303 (100 mg L^{-1} GO)	10.5 ± 0.4	
daf-2 intestine RNAi (control)	22.3 ± 0.8	$P < 0.01$
daf-2 intestine RNAi (100 mg L^{-1} GO)	20.9 ± 1.2	$P < 0.01$
daf-16 intestine RNAi (control)	10.3 ± 0.5	$P < 0.01$
daf-16 intestine RNAi (100 mg L^{-1} GO)	6.7 ± 0.6	$P < 0.01$
VP303 (control)	13.1 ± 0.4	
VP303 (100 mg L^{-1} GO)	10.4 ± 0.5	
age-1 intestine RNAi (control)	22.3 ± 0.9	$P < 0.01$
age-1 intestine RNAi (100 mg L^{-1} GO)	20.4 ± 0.6	$P < 0.01$
daf-18 intestine RNAi (control)	10.1 ± 0.6	$P < 0.01$
daf-18 intestine RNAi (100 mg L^{-1} GO)	7.1 ± 0.6	$P < 0.01$
VP303 (control)	12.7 ± 0.6	
VP303 (100 mg L^{-1} GO)	10.1 ± 0.5	
akt-1 intestine RNAi (control)	16.4 ± 1.1	$P < 0.01$
akt-1 intestine RNAi (100 mg L^{-1} GO)	15.2 ± 0.9	$P < 0.01$
akt-2 intestine RNAi (control)	16.9 ± 0.8	$P < 0.01$
akt-2 intestine RNAi (100 mg L^{-1}	16.1 ± 0.9	$P < 0.01$

GO)		

Table S6 | Effects of intestinal RNAi of *sod-3* gene on GO toxicity on lifespan in nematodes

Treatment	Mean lifespan (day)	Significance (compared with VP303)
VP303 (control)	13.6 ± 0.8	
VP303 (100 mg L^{-1} GO)	11.3 ± 0.5	
sod-3 intestine RNAi (control)	13.7 ± 0.7	NS
sod-3 intestine RNAi (100 mg L^{-1} GO)	8.9 ± 0.7	$P < 0.01$

NS, no significance.

Table S7 | Genetic interaction between SOD-3 and DAF-16 in regulating GO toxicity on longevity in nematodes

Treatment	Mean lifespan (day)	Significance (compared with wild-type)
Wild-type (control)	15.5 ± 0.5	
Wild-type (100 mg L^{-1} GO)	12.3 ± 0.4	
Ex(P*ges-1-daf-16*) (control)	22.7 ± 0.6	*P* < 0.01
Ex(P*ges-1-daf-16*) (100 mg L^{-1} GO)	21.9 ± 0.8	*P* < 0.01
Wild-type	14.9 ± 0.8	
Ex(P*ges-1-daf-16*)	27.6 ± 0.6	*P* < 0.01
sod-3(gk235)	14.7 ± 0.9	NS
Ex(P*ges-1-daf-16*);*sod-3(gk235)*	26.1 ± 0.5	*P* < 0.01
Wild-type (100 mg L^{-1} GO)	12.6 ± 0.5	
Ex(P*ges-1-daf-16*) (100 mg L^{-1} GO)	22.4 ± 0.9	*P* < 0.01
sod-3(gk235) (100 mg L^{-1} GO)	8.6 ± 0.6	*P* < 0.01
Ex(P*ges-1-daf-16*);*sod-3(gk235)* (100 mg L^{-1} GO)	9.5 ± 0.8	*P* < 0.01

NS, no significance.

Table S8 | Information for RT-PCR primers

Gene	Forward primer	Reverse primer
tba-1	TCAACACTGCCATCGCCGCC	TCCAAGCGAGACCAGGCTTCAG
daf-2	ATGTGGCGTGAGAATGAA	AGCCGAACACGAACAACA
age-1	ATGGAAACCGCCGAGTGT	ATTGGCAGTCGGTTCAGG
pdk-1	TTCAGAGCCGTCAACCAG	GCTCACTTGCTCGGCTTT
sgk-1	AAGACTGTTGACTGGTGGTG	AGACGAAGTGGCTGGTTG
akt-1	GGACAACCGTTTCCTGAG	GACGAACTTCTGCCGACT
akt-2	ATCAGCCGTTACCAGAGC	AAGGTTCCTTGACCGAGA
daf-18	ATCATCATCCGCCGAGTC	ACCGTTGAGTCCTCCATC
daf-16	CGTTTCCTTCGGATTTCA	ATTCCTTCCTGGCTTTGC
